# Double aneuploidy mosaicism involving chromosomes 18 and 21 in a neonate

**DOI:** 10.1186/s13039-021-00578-7

**Published:** 2022-01-24

**Authors:** Christina Mendiola, Veronica Ortega, Allison Britt, Rafael Fonseca, Gopalrao Velagaleti

**Affiliations:** 1 Department of Pathology and Laboratory Medicine, UT Health –San Antonio, 7703 Floyd Curl Drive, Mail Code 7750, San Antonio, TX 78229 USA; 2 Department of Pediatrics, University of Texas Medical Branch, Galveston, TX USA

**Keywords:** Double aneuploidy, Trisomy 18, Trisomy 21, Non-disjunction, SNP microarray

## Abstract

**Background:**

Double aneuploidy is common, especially in products of conception, frequently involving a combination of a sex chromosome and an acrocentric chromosome. Double autosomal trisomies are rare with only five cases reported. Double aneuploidy mosaicism involving two different cell lines is rarer with only three cases reported.

**Case presentation:**

We report a fourth case of double aneuploidy mosaicism on a baby. Results of a 24-h preliminary chromosome analysis at birth showed a mosaic karyotype, 47,XX,+18[15]/47,XX,+21[8]/48,XX,+21,+mar[7]. Reflex testing to SNP microarray with the same sample collected at birth showed gain of a 77.9 Mb region on chromosome 18 and gain of a 32.5 Mb region on chromosome 21. Microarray did not show any other copy number variants indicating that the marker chromosome may not contain any euchromatic material. A repeat chromosome analysis at 1-year of age showed a mosaic karyotype, 47,XX,+18[76]/47,XX,+21[4] with loss of the marker cell line.

**Conclusion:**

Based on our results, we propose that the mosaic double autosomal trisomy in our case was due to two independent non-disjunction events in a normal zygote very early during embryogenesis.

## Background

Double aneuploidy is common, especially in products of conception, frequently involving a sex chromosome and an acrocentric chromosome [1–3]. Double autosomal trisomies in live born infants are rare with only five cases reported having combinations of chromosomes 8 and 14; 8 and 21; 13 and 18; 13 and 21; and 18 and 21 [4]. Double aneuploidy mosaicism involving two different aneuploidy cell lines is even rarer in live born infants with only three cases previously reported [5–7]. Several mechanisms propose to explain the origin of mosaic double aneuploidy including two independent non-disjunction events in a normal zygote, two independent anaphase lag events in a non-mosaic double aneuploidy zygote and independent trisomy rescue of different trisomies in different cell lines [7]. We report a case of double aneuploidy mosaicism on a baby referred for cytogenetic testing due to a positive prenatal quad and non-invasive prenatal screening test for trisomy 21.

## Material and methods

### Case presentation

A 36^4/7^ week preterm, appropriate for gestational age, baby girl was admitted to neonatal intensive care unit because of atrioventricular canal defect and a possible jejunal atresia. She was born to a 41-year-old G2P1A1 mother. The prenatal history is unremarkable with both parents not showing any dysmorphic or other clinical features and both parents reporting no relevant clinical history or congenital abnormalities. Fetal ultrasound at 29 weeks showed an atrial septal defect, pericardial effusion and a small bowel dilatation with possible double bubble sign. Quad screen was positive for trisomy 21 and a subsequent non-invasive prenatal screening was positive for trisomy 21. Mother declined amniocentesis.

At birth, the baby weighed 2470 g and her length was 46 cm with a head circumference of 34 cm with Apgar scores of 9 and 9 at 1 and 5 min, respectively. The proband was alert and showed no obvious dysmorphic features suggestive of trisomy 21 or 18. Physical examination was unremarkable. Exploratory laparotomy was performed to repair the jejunal atresia and a subsequent exploratory laparotomy was performed to repair a right inguinal hernia and gastrostomy tube placement with Nissen fundoplication. Ophthalmology evaluation showed Peter’s anomaly with left cataract. Cardiac evaluation revealed complete common atrioventricular septal defect (AVSD) with large primum atrial septum defect (ASD), large endocardial cushion ventricular septal defect (VSD), large endocardial cushion VSD, biventricular hypertrophy and mild AV valve regurgitation. No evidence of dilated or hypertrophic cardiomyopathy noted. The baby was transferred to a tertiary neonatal intensive care unit for further care.

Follow-up evaluation in the Genetics clinic at 8 months of age revealed AVSD, hypotonia, low-set posteriorly rotated ears, high arched palate, micrognathia, mild rocker-bottom feet, and cataract. Renal ultrasound revealed bilateral increased renal echogenic parenchyma.

Brain MRI at 13 months of age showed moderate to severe central white matter volume loss with secondary thinning of a normally formed corpus callosum and associated moderate ex vacuo dilatation of the lateral ventricles. There was no evidence of hydrocephalus. Secondary associated pontine and brachium pontis volume loss was seen. Moderate enlargement of the subarachnoid spaces overlying the bilateral cerebral convexities with trace subdural fluid collection overlying the left frontal lobe without associated blood products was also observed. At 2.5 years of age, the baby had cardiac surgery. No further cardiac complications have occurred and she continues to follow with cardiology. At 4 years of age, she follows with a multispecialty team including gastroenterology for constipation controlled with Miralax and feeding difficulty. She is primarily G tube fed, but can take some liquids and purees by mouth. She follows with ophthalmology for her left cataract. Development is delayed with gross motor skills at the 13–15 month level, and speech skills at the 7–11 month level. She receives regular monitoring for Down syndrome as recommended by the American Academy of Pediatrics.

### Cytogenetic studies

Peripheral blood sample from the baby was cultured with RPMI-1640 medium and harvested using standard operating procedures. Routine chromosome analysis (RCA) was performed on G-banded metaphases at 400 and 550 band resolution using CytoVision® software 7.6 (Leica Biosystems, Buffalo Grove, IL).

### Fluorescence in situ hybridization (FISH) studies

FISH studies were performed with Abbott Aneyvysion probe set (Abbott Molecular, Des Plains, IL). This probe set includes a combination of chromosome 18 (D18Z1 labeled in spectrum aqua), chromosome X (DXZ1 labeled in spectrum green) and chromosome Y (DYZ3 labeled in spectrum orange) and a second combination of chromosomes 13 (RB1 gene labeled in spectrum green) and chromosome 21 (D21S259, D21S341 and D21S342 labeled in spectrum orange). Slide preparation, probe hybridization, and post-hybridization washing was performed using standard procedures. Slides were analyzed using CytoVision® software 7.6. For interphase analysis, a minimum of 100 nuclei were scored, and for metaphase analysis, a minimum of 10 metaphases were scored.

### High-resolution chromosomal microarray studies

Chromosome microarray studies were carried out using Affymetrix CytoScan HD microarray. The Affymetrix CytoScan® HD Assay utilizes a high density combined CGH and SNP array platform, which assesses approximately 2,696,550 markers, including approximately 750,000 SNP markers. Each oligonucleotide is approximately 25 base pairs long. Intragenic probe spacing is approximately 1 probe every 880 base pairs and intergenic probe spacing is approximately 1 probe every 1700 base pairs. To perform the assay, gDNA is digested with the Nsp1 restriction enzyme and digested DNA is then ligated to Nsp1 adapters. The ligation product is amplified via polymerase chain reaction (PCR) to produce amplicons in the 200–1100 bp range. The amplicons are purified and digested with DNAse I to produce 25–125 bp fragments. The fragments are end-labeled with a modified biotinylated base and the sample is hybridized to the array. The array is washed and stained with a streptavidin-coupled dye and a biotinylated anti-streptavidin antibody. The array is scanned with the GeneChip Scanner and the signal intensity for each marker is assessed. Using the Chromosome Analysis Suite (ChAs 3.0) software, the signal for the sample is then compared to a reference set, which is based on the average of over 400 samples. Differences in signal between the sample and reference are expressed as a log2 ratio and represents relative intensity for each marker. A discrete copy number value is determined from the relative intensity data and displayed. Genotype information for the SNP markers is visualized with the Allele Track [8].

## Results

**Fig. 1:**
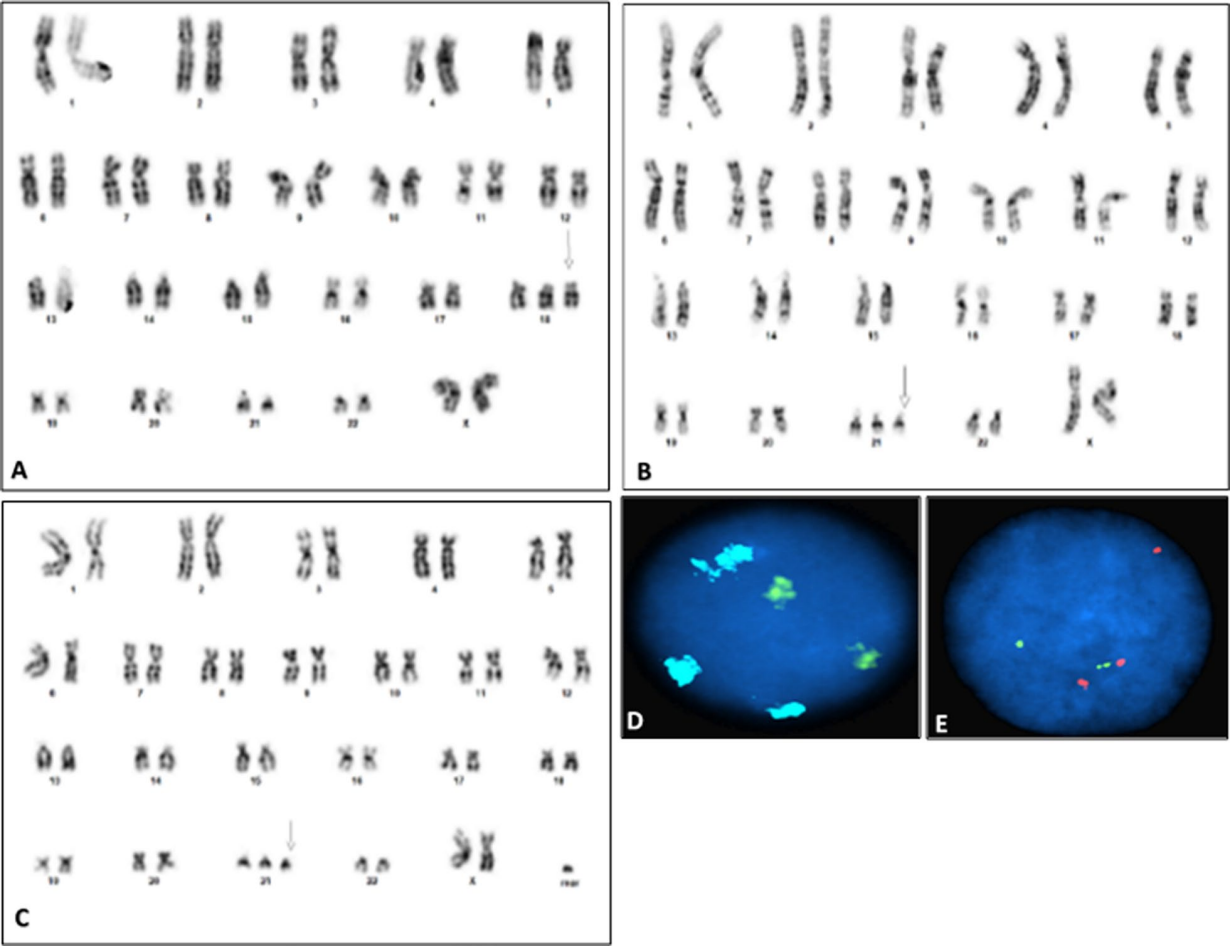
GTG-banded karyotype at birth showing **A** trisomy 18, **B** trisomy 21 and **C** trisomy 21,+mar. FISH study at birth showing interphase nuclei with **D** trisomy 18 in AQUA and chromosome X in green and **E** trisomy 21 in orange

Cytogenetic analysis from peripheral blood at birth showed a female karyotype with 3 different cell lines. The predominant cell line with 50% of the cells showed trisomy 18, followed by a second cell line with 26.7% of the cells showing trisomy 21 and the third cell line with 23.3% of the cells showing trisomy 21 and an unidentified marker chromosome (Fig. 1A–C; Table 1). The marker chromosome was smaller than a G group chromosome in size and appeared to contain a centromere. The karyotype was interpreted as 47,XX,+18[15]/47,XX,+21[8]/48,XX,+21,+mar[7] [9].

**Table 1:** Percentage of trisomic cells from chromosome, microarray and FISH analyses

Test	% of cells with + 18	% of cells with + 21	% of cells with + 21 and + mar	Normal cells
RCA (at birth)	50	26.7	23.3	0%
Microarray (at birth)	67	30	0	3%
FISH (at birth)	66	21.5	0	12.5%
RCA (at 1 year)	95	5	0	0%

FISH studies on the same sample collected at birth with Abbott Aneuvysion probes showed 66% of the interphase cells with 3 signals for chromosome 18, 21.5% of the cells showed trisomy 21 and the remaining cells showed normal signal pattern for both chromosomes 18 and 21 (Fig. 1D, E; Table 1). This result suggested that the marker chromosome did not originate from any of the chromosomes within this probe cocktail. Metaphase analysis also confirmed that the marker chromosome did not show any signals for any of the chromosomes in the cocktail.

**Fig. 2:**
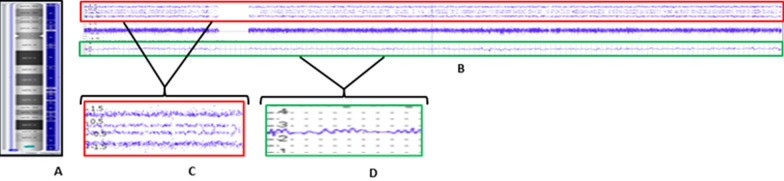
Karyoview at birth using Affymetrix CytoScan® HD array (ChAS 3.0), showing gains along the length of chromosome 18 (**A**). Segment view of chr.18 showing gain from 18p11.32 to 18q23 (**B**). Allele peaks showing 4 tracks (**C**) and Smooth signal showing gain (**D**)

**Fig. 3:**
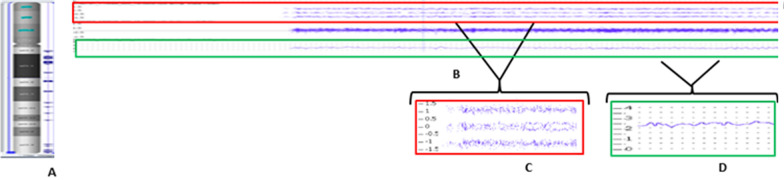
Karyoview at birth using Affymetrix CytoScan® HD array (ChAS 3.0), showing gains along the length of chromosome 21 (**A**). Segment view of chr.21 showing gain from 21q11.2 to 21q22.3 (**B**). Allele peaks showing 4 tracks (**C**) and Smooth signal showing gain (**D**)

Results of high-resolution SNP microarray study on the same sample collected at birth showed a 77.9 Mb gain on chromosome 18 from 18p11.32 to 18q23 (Fig. 2A–D) and a 32.5 Mb gain on chromosome 21 from 21q11.2 to 21q22.3 (Fig. 3A–D). The microarray analysis showed that the trisomy 18 cell line is predominant with 67% of cells while the trisomy 21 cell line represents approximately 30% of cells (Table 1). Microarray did not show any other copy number variants indicating that the marker chromosome may not contain any euchromatic material. Additional targeted microarray analysis or M-FISH studies are required to explore the source of marker chromosome.

A repeat chromosome analysis at 1 year of age on the peripheral blood lymphocytes showed only 2 cell lines, trisomy 18 and trisomy 21 with the karyotype 47,XX,+18[76]/47,XX,+21[4] [9].

## Discussion

This is the fourth case of a mosaic autosomal trisomy involving chromosomes 18 and 21 in a live born baby. Unlike previously published cases showing only phenotypic features of trisomy 21, our proband showed features consistent with both trisomy 21 and trisomy 18 [5–7]. The phenotypic features such as AVSD, hypotonia, and low-set posteriorly rotated ears, can be attributed to both trisomy 21 and trisomy 18. Features like high arched palate, micrognathia, mild rocker-bottom feet, and cataract are more consistent with trisomy 18. In all the previously published cases, the trisomy 21 cell line is predominant in lymphocytes (≥ 80%) with trisomy 18 being the minor cell line, while trisomy 18 cell line was not observed in skin fibroblasts in the case reported by Jenkins et al. [6]. This predominance of trisomy 21 cell line may explain the correlation with the phenotypic features of trisomy 21. The discrepancy with reference to lack of trisomy 18 features in all published cases could be due in part also to tissue heterogeneity. In our proband, on the contrary, the trisomy 18 cell line is the predominant cell line and that may explain the combined features of both trisomy 21 and trisomy 18. Additional studies of other tissues to see if our proband has a different genetic composition in different tissues would contribute to this investigation. However, given the extensive surgical and medical issues with our proband, any additional studies, although contemplated, are not possible at this time. It is also possible that with aging, our proband will show clinical features more consistent with one of the trisomies or combination of both, depending on the tissue composition of each trisomic cell line. This age related phenotypic expression could be one of the explanations for lack of obvious phenotypic features at birth in our proband.

The proportion of various cell lines in the peripheral blood tissue in our proband (Table 1) is consistent across all the methods of analysis. While chromosome analysis and FISH were performed on cultured cells, uncultured blood was used for microarray analysis. Our results may suggest that there is no selection bias for any trisomic cell line in culture. As is known with mitotic instability of small centromeric super numerary marker chromosomes, the marker chromosome that was originally seen at birth disappeared in our proband at age 1 year [10, 11]. The percentage of trisomic cell lines appears to be also unstable with age as the trisomy 18 cell line became even more predominant while the trisomy 21 cell line appears to be significantly reduced at age 1 year. While there was no selection bias for any particular trisomic cell line in vitro at birth, trisomy 18 cell line appears to have selective advantage for proliferation at age 1 year as seen in the repeat chromosome analysis on peripheral blood.

Three different mechanisms were suggested to account for the mosaic double aneuploidy, (1) non-disjunction of both chromosomes 18 and 21; (2) anaphase lag of chromosomes 18 and 21; and (3) non-disjunction and anaphase lag of either chromosome 18 or 21 [7]. Since the net result of anaphase lag is gain of a chromosome by one daughter cell while the other daughter cell will have normal diploid chromosome complement, we propose that anaphase lag is not likely to be the mechanism in our case for the double mosaic aneuploidy. On the other hand, the net result of a non-disjunction event is gain of a chromosome in one daughter cell while it’s complementary daughter cell loses the same chromosome, we hypothesize that non-disjunction most likely is the cause of double mosaic aneuploidy in our case.

**Fig. 4:**
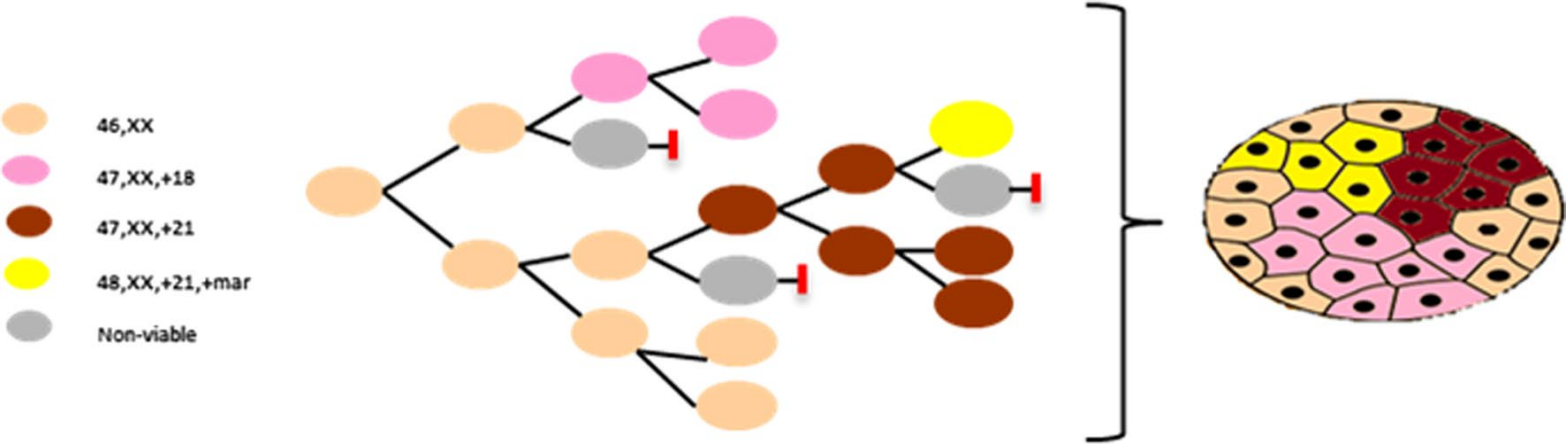
Hypothesized diagram showing mechanism of origin of double mosaic aneuploidy

Although routine chromosome analysis did not show any normal cells in our case, FISH analysis showed about 12.5% of the cells with normal signal pattern indicating that there are normal diploid cells. Based on this result, we propose that two independent post-fertilization non-disjunction events in an otherwise normal diploid zygote may have resulted in the double aneuploidy in our case (Fig. 4). In our model, during normal development of the fertilized diploid zygote, a mitotic non-disjunction during early embryogenesis resulted in trisomy 18 in some cells and monosomy 18 in other cells derived after the error. Since monosomy 18 is lethal, the cell population with this chromosome complement did not survive resulting in trisomy 18 cells and normal diploid cells. A second non-disjunction event during later embryogenesis resulted in trisomy 21 and monosomy 21 cells with monosomy 21 cells not proliferating further. A 3rd non-disjunction error in the trisomy 21 cell population resulted in cells with trisomy 21 and marker chromosome. This ultimately resulted in four separate cell populations, a normal diploid cell line, trisomy 18 cell line, trisomy 21 cell line, and trisomy 21 with marker cell line. Since the error resulting in trisomy 18 probably occurred as the first error, there were more trisomy 18 cells.

## Conclusion

One of the major drawbacks of our study is our inability to study other tissues to determine if there is heterogeneity with reference to mosaicism in various tissues. Even with this limitation, our case represents only the 4th case in literature of double mosaic aneuploidy in a live born baby with trisomy 18 and trisomy 21.

## Data Availability

All relevant data and material is included in this publication.

## Abbreviations

ASVD: Atrioventricular septal defect
ASD: Atrial septal defect
FISH: Fluorescence in situ hybridization
RCA: Routine chromosome analysis
SNP: Single nucleotide polymorphisms

## References

[1] Reddy KS (1997). Double trisomy in spontaneous abortions. Hum Genet.

[2] Diego-Alvarez D, Ramos-Corrales C, Garcia-Hoyos M, Bustamante-Aragones A, Cantalapiedra D, Diaz-Recasens J, Vallespin-Garcia E, Ayuso C, Lorda-Sanchez I (2006). Double trisomy in spontaneous miscarriages. Cytogenet Mol Approach Hum Reprod.

[3] Micale M, Insko J, Ebrahim SA, Adeyinka A, Runke C, Van Dyke DL (2010). Double trisomy revisited—a multicenter experience. Prenat Diagn.

[4] Huijsdens-van Amserdam K, Barge-Schaapveld DQCM, Mathijssen IB, Alders M, Pajkrt E, Knegt AC (2012). Prenatal diagnosis of a trisomy 7/trisomy 13 mosaicism. Mol Cytogenet.

[5] Marks JF, Wiggins KM, Spector BJ (1967). Trisomy 21-trisomy 18 mosaicism in a boy with clinical Down’s syndrome. J Pediatr.

[6] Jenkins MB, Kriel RL, Boyd C, Barnwell B (1978). Trisomy 21 with 47,+18 lymphocyte cell line: double mitotic non-disjunction. J Med Genet.

[7] Thomas IM, Sayee R, Shavanthi L, Hegde S (1994). Trisomy 18 and trisomy 21 mosaicism in a Down’s syndrome patient. J Med Genet.

[8] Webb BD, Scharf RJ, Spear EA, Edelmann J, Stroustrup A (2015). Evaluation of the Affymetrix Cytoscan® Dx assay for developmental delay. Expert Rev Mol Diagn.

[9] McGowan-Jordan J, Simons A, Schmid M (eds). An international system for human cytogenetic nomenclature, S. Karger, Basel.

[10] Hussein SS, Kreskowski K, Ziegler M, Klein E, Hamid AB, Kosyakova N, Volleth M, Leihr T, Fan X, Piaszinski K (2014). Mitotic stability of small supernumerary marker chromosomes depends on their shape and telomeres—a long term in vitro study. Gene.

[11] Spittel H, Kubek F, Kreskowski K, Ziegler M, Klein E, Hamid AB, Kosyakova N, Radhakrishnan G, Junge A, Kozlowski P, Schulze B, Martin T, Huhle D, Mehnert K, Rodriguez L, Ergun MA, Sarri C, Militaru M, Stipoljev F, Tittelbach H, Vasheghani F, de Bello CM, Hussein SA, Fan X, Volleth M, Liehr T (2014). Mitotic stability of small supernumerary marker chromosomes: a study based on 93 immortalized cell lines. Cytogenet Genome Res.

